# Unilateral Retinitis Pigmentosa Associated with Possible Ciliopathy and a Novel Mutation

**DOI:** 10.3390/clinpract12040053

**Published:** 2022-07-05

**Authors:** Doaa Milibari, Moustafa Magliyah, Valmore A. Semidey, Patrik Schatz, Hani B. ALBalawi

**Affiliations:** 1 Vitreoretinal Division, King Khaled Eye Specialist Hospital, Riyadh 11462, Saudi Arabia; doaamilibari@gmail.com (D.M.); asemidey@hotmail.com (V.A.S.); patrik.schatz@med.lu.se (P.S.); 2 Department of Ophthalmology, King Abdullah Medical City, Makkah 56757, Saudi Arabia; 3 Ophthalmology Department, Prince Mohammed Medical City, Sakakah 11451, Saudi Arabia; mussam08@gmail.com; 4 Department of Ophthalmology, Clinical Sciences, Skane University Hospital, Lund University, Lund 11462, Sweden; 5 Ophthalmology Division, Surgery Department, Faculty of Medicine, University of Tabuk, Tabuk 71491, Saudi Arabia

**Keywords:** URP, ffERG, retinal dystrophy, *AGBL5*, RPGR, ciliopathy

## Abstract

Unilateral retinitis pigmentosa (URP) is a rare retinal dystrophy. We describe the clinical course of two patients with (URP) unilateral retinitis pigmentosa confirmed by genetic testing, indicating ciliary dysfunction. Methods: The methods used in this study included a detailed ophthalmic examination, multimodal retinal imaging, Goldmann visual fields, full-field electroretinography (ffERG) and targeted next-generation sequencing. Results: A 32-year-old female (patient 1) and 65-year-old male (patient 2) were found to have URP. ffERG showed a non-recordable response in the affected eye and a response within normal limits in the fellow eye of patient 1, while patient 2 showed non-recordable responses in the apparently unaffected eye and a profound reduction in the photopic and scotopic responses in the affected eye. Next-generation sequencing revealed novel compound heterozygous c.373 C>T (p.Arg125Trp) and c.730-22_730-19dup variants in *AGBL5* in patient 1, and a novel hemizygous c.1286 C>T (p.Pro429Leu) in patient 2; both gene mutations were 0%. Segregation analysis was not possible for either of the mutations. Conclusion: This report expands the clinical and molecular genetic spectrum of URP.

## 1. Introduction

Retinitis pigmentosa includes a group of inherited retinal dystrophies that manifest as the progressive degeneration of rod and cone photoreceptors. Retinitis pigmentosa is typically a bilateral disease. Affected patients usually complain of night blindness, peripheral visual field constriction and, in advanced cases, severe visual loss and blindness. Unilateral retinitis pigmentosa (URP) is a rare retinal dystrophy, which has been estimated to be present in approximately 5% of retinitis pigmentosa cases [1]. URP was initially presumed to be X-linked and it was first described in 1948 by Dresiler. Fewer than 100 cases have been reported in the literature [2]. Two possible genetic mechanisms have been suggested to play a role in asymmetrical RP or URP, namely, mosaicism and somatic mutations during embryogenesis. However, the exact pathogenesis and genetic mechanism are currently unclear [1,3,4,5].

Genetic associations with URP have recently been reported, including mutations in *RP1* [6], *RPGR* [4], *CLRN1* [7] and *USH2A* [5]. These have only been described in a handful of URP cases. Unilateral pigmentary retinopathy (pseudo retinitis pigmentosa) can also arise from a variety of conditions such as ocular trauma, inflammation, retained metallic intraocular foreign body, infections (toxoplasmosis, toxocariasis, syphilis, Lyme disease) and diffuse unilateral subacute neuroretinitis (DUSN), drug toxicity (chloroquine and chlorpromazine), autoimmune retinopathy and cancer-associated retinopathy (CAR) [4]. Key points that help in the differentiation of URP from common differential diagnoses are summarized in Table 1.

Retinal ciliopathy was initially observed in patients with X-linked retinitis pigmentosa and Usher syndrome. Thus far 64 genes have been reported to be associated with RP in the literature, and at least 18 of these genes encode proteins that localize in the cilia: *ARL6, BBS1, BBS9, C2ORF71, C8ORF37, CLRN1, FAM161A, MAK, TTC8, TULP1, USH2A* and *CEP290, RP1, TOPORS* and *RP1L1, OFD1, RP2,* and *RPGR* [8].

There is a close association between genes associated with URP and ciliopathy. The RP1 and RPGR, and the proteins encoded by genes associated with Usher syndrome are found in the connecting cilium of both the cone and rod photoreceptors.

As per RP, retinal ciliopathies are often characterized by severe and progressive loss of the ellipsoid zone line and gradual constriction of the remaining preserved outer retina, and this can be followed using a serial autofluorescence imaging, which shows a constricting hyperautofluorescent ring over time in the posterior pole [8,9].

In this study, we report the ocular findings and workup, including molecular genetic testing in two patients with URP with possible ciliopathy.

## 2. Materials and Methods

Informed consent was obtained, and the study was approved by an institutional review board at King Khaled Eye Specialist Hospital.

Ophthalmic examination included multimodal retinal imaging such as fundus photos, autofluorescence (Optos TM (Dunfermline, Scotland, UK), spectral domain optical coherence tomography (SD-OCT, Heidelberg Engineering, Heidelberg Germany), Goldmann visual fields (Goldmann perimeter, Haag-Streit, Switzerland) [10], and full-field electroretinography (ffERG).

ffERG (Nicolet Biomedical Instruments, Madison, WI, USA) was obtained as follows, in dark-adapted and light-adapted state according to International Society for Clinical Electrophysiology of Vision (ISCEV) standards with a few modifications as described by us before [11,12].

The blood samples were taken from both patients and genetic testing was performed at Bioscientia Human Genetics laboratory (Bioscientia, Ingelheim, Germany). Genomic DNA was fragmented, and the coding axons of the analyzed genes as well as the corresponding exon–intron boundaries were enriched using the Roche/NimbleGen sequence capture approach, amplified and sequenced simultaneously by Illumina technology system (next-generation sequencing, NGS). The target regions were sequenced with an average coverage of 1049-fold. NGS data analysis was performed using bioinformatic analysis tools as well as JSI Medical Systems software. Identified variants and indels ware filtered depending on their allele frequency focusing on rare variants with a minor allele frequency (MAF) of 1% or less. In silico analysis of identified variants with regard to functional relevance, conservation and splice effects was performed using bioinformatic prediction program. Classification of variants was performed based on ACMG guidelines [13]. Putatively pathogenic differences between the wildtype sequence (human reference genome according to UCSC Genome Browser. hg19, GRCh37) and the patients’ sequences were assessed. Variants were verified using polymerase chain reaction (PCR) amplification followed by conventional Sanger sequencing.

The panel for autosomal recessive (AR) RP was used in patient 1, while the panel for cone–rod dystrophy (CRD)/cone dystrophy (CD)/macular dystrophy (MD) for patient 2.

ARRP panel includes: ABCA4, ABHD12, ACACB, ADGRA3, ADIPOR1, AGBL5, ARL2BP, ARL6, ASRGL1, BBS4, BEST1, C2ORF71, C8ORF37,CC2D2A, CDH16, CERKL, CLRN1, CNGA1, CNGB1, CRB1, CYP4V2, DHDDS, DHX38, DNAJC17, EMC1, EYS, FAM61A, FLVCR1, GNPTG, GNS, GRID2, HGSNAT, IDH3B, IFT140, IFT172, IMPG1, IMPG2, KIAA1549, KIZ, LAMA1, LRAT, MAK, MERTK, MFSD8, MPDZ, MTTP, MVK, NEK2, NEUROD1, NR2E3, NRL, PDE6A, PDE6B, PED6G, PLA2G5, PNPLA6, PRCD, PROM1, PFPR31, PRPH2, RBPH2, PBP3, PBP4, RDH11, RDH12, RDH5, RPGR, RHBDD2, RHO, RLBP1, RP1, RPE65, RPGRIP1, SAG, SLC7A14, SPATA7,TRNT1,TTC8, TPPA, TUB, TULP1,USH1C, USH2A, WDR19, ZNF513.

CRD/CD/MD panels include: ABCA4, ACBD5, ADAM9, AIPL1, BEST1, C1OTNF5, C21ORF2, C8ORF37, CABP4, CANCA1F, CANCA2D4, CDHR1, CERKL, CFH, CNGA3, CNGB3, CNNM4, CRX, DRAM2, ELOVL4, FBLN5, FSCN2, GUCA1A, GUCY2D, HMCN1, ITM2B, KCNV2, MERTK, MFSD8, MT-ATP6, MT-TL1, PCYT1A, PDE6C, PITPNM3, PLK4, POC1B, PRDM13, PROM1, PRPH2, RAB28, RAX2, RDH5, RGS9, RGS9BP, RIMS1, RP1L1, RPGR, RPGRPIP1, SEMA4A, TEAD1, TIMP3, TUBGGCP4, TUBGCP6, UNC119, WASF3.

## 3. Results

### 3.1. Patient 1

A 32-year-old female presented four years ago with a unilateral decrease in vision in the left eye that she had had for a number of years. There was no family history of blindness or inherited eye diseases and her parents were first cousins.

**Figure 1:**
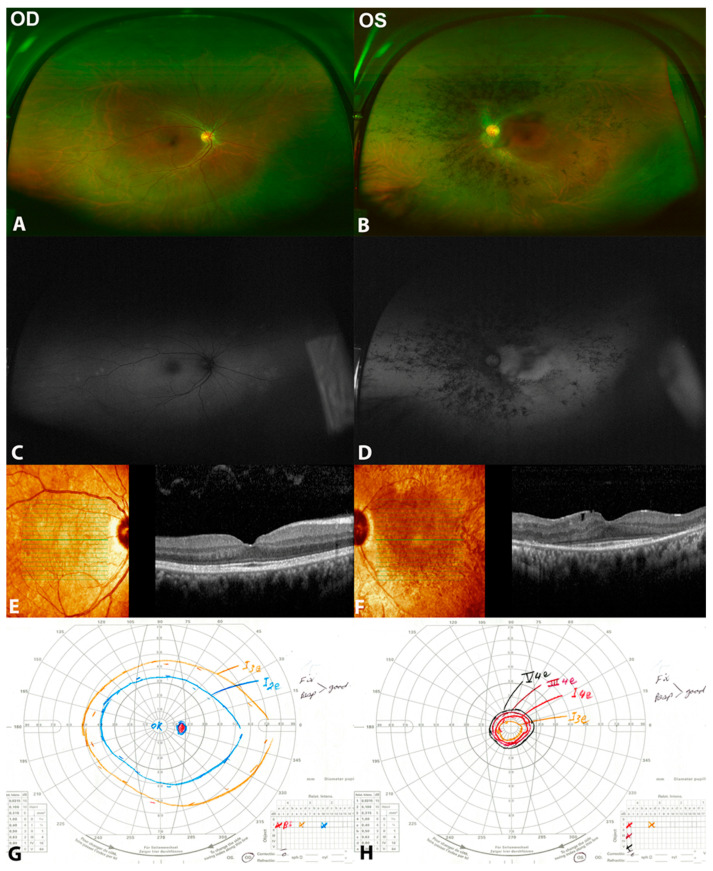
Clinical and multimodal findings of (patient 1) a 32-year-old female with unilateral retinitis pigmentosa (URP) in the left eye with two heterozygous variants c.373 C>T (p.Arg125Trp) and c.730-22_730-19 dup in *AGBL5*. (**A**,**B**) are color fundus photos showing normal right eye, while they show midperipheral bone spicules more condensed nasally, attenuated blood vessels, and pale disc. (**C**,**D**) are fundus autofluorescence photos showing normal right eye and hypoautofluorescence corresponding to the bone spicules, hyperautofluorescence at the macular area with reserved foveal autofluorescence in the left eye. (**E**,**F**) are spectral domain optical coherence tomography (SD-OCT) showing normal right eye. However, there is a peripheral loss of the ellipsoid zone with foveal sparing in the left eye. (**G**,**H**) are Goldmann visual fields showing normal visual field in the right eye and tunnel visual field in the left eye.

She denied a previous history of ocular trauma, surgery and ocular inflammation. Her medical and drug history were unremarkable. The patient denied any neurological or hearing problems. She had best-corrected visual acuity (BCVA) of 20/20 and 20/40 in the right and left eye, respectively. Anterior segment examination of both eyes, as well as fundus examination of the right eye were unremarkable. Fundus examination of the left eye revealed midperipheral bone spicules, attenuated blood vessels and a waxy pallor of the optic disc (Figure 1).

**Figure 2:**
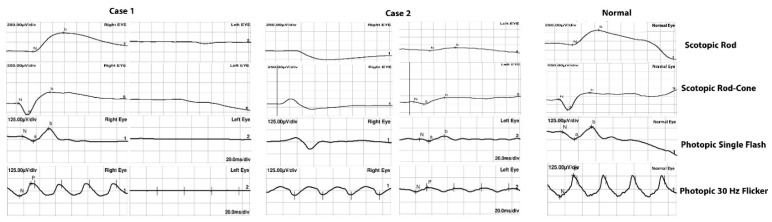
Full-field electroretinogram showing normal responses of the right eye and undetectable responses of the left eye in patient 1 a 32-year-old female with unilateral retinitis pigmentosa (URP) in the left eye with two heterozygous variants c.373 C>T (p.Arg125Trp) and c.730-22_730-19 dup in *AGBL5*. Patient 2, a 65-year-old male with URP in the left eye, with *RPGR* missense variant c.1286 C>T (p.Pro429Leu), showed unrecordable responses of the right eye, while responses of the left eye were profoundly reduced.

Fundus autofluorescence revealed hypoautofluorescence corresponding to the bone spicules and hyperautofluorescence at the macular area with preserved foveal autofluorescence. SD-OCT of the right eye was unremarkable, while the left eye showed the loss of the parafoveal ellipsoid zone. The subfoveal ellipsoid zone was minimally affected. ffERG showed responses within normal limits in the right eye and non-recordable responses in the left eye (Figure 2). The Goldman visual field was normal in the right eye, while the left revealed a severely constricted field with a remaining small central island. Next-generation sequencing for autosomal recessive retinal dystrophies identified two heterozygous variants, which were initially classified as variants of uncertain significance (VUS) in the *AGBL5* gene (NM_021831.5), c.373 C>T (p.Arg125Trp) and c.730-22_730-19 dup.

Neither of the *AGBL5* variants, c.373 C>T (p.Arg125Trp) or c.730-22_730-19 dup, have been described in the literature so far. The allele frequency of this variant has not been documented in the normal population (GnomAD v2.1.1 controls; accessed on 15 August 2020). The variant c.730-22_730-19 may lead to a splice defect. One out of three bioinformatic in silico programs predict a significant alteration of mRNA splicing due to the activated cryptic splice site. Segregation analysis for parents and other family members was not possible. The patient had stable vision upon examination in both eyes during five years of follow-up visits with a stable average central macular thickness on SD-OCT (Table 2).

### 3.2. Patient 2

A 65-year-old male presented with a unilateral gradual decreased vision of the left eye associated with photopsia that he had had for more than 20 years.

Past medical history included diabetes, hypertension and nasopharyngeal carcinoma treated with chemotherapy (carboplatin and docetaxel) 4 years ago.

Past ocular history revealed a trauma to the left eye at the age of 5 years with no clear relation of visual loss at that time. There was no family history of blindness or inherited retinal diseases and no paternal consanguinity. The patient denied any neurological or hearing problems.

**Figure 3:**
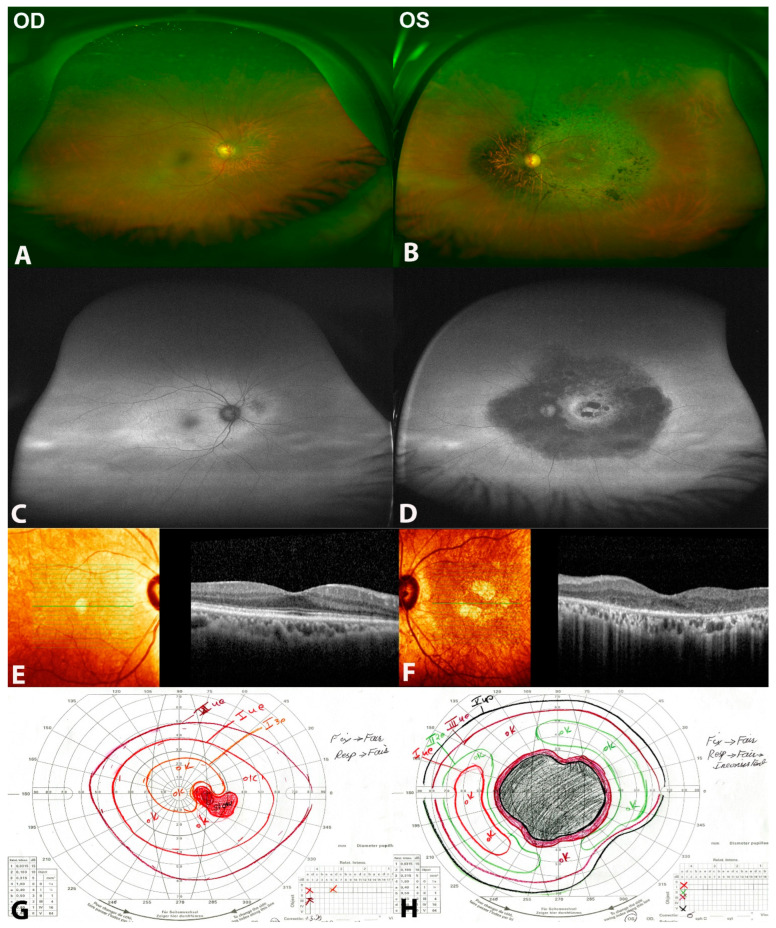
Clinical and multimodal findings of (patient 2) a 65-year-old male with URP in the left eye with the *RPGR* missense variant c.1286 C>T (p.Pro429Leu). (**A**,**B**) are color fundus photos showing chorioretinal atrophic changes nasal to the optic disc in the right eye, while showing midperipheral bone spicules, localized retinal thinning nasal to the optic disc, attenuated blood vessels, waxy pallor of the optic disc and atrophic macula in the left eye. (**C**,**D**) are fundus autofluorescence photos showing a ring of hyperautofluorescence around the optic disc in the right eye and hypoautofluorescence in the posterior pole around the disc and macula, hyperautofluorescent ring at the macular area with patches of well demarcated hypoautofluorescence in the left eye. (**E**,**F**) show SD-OCT of the right eye as normal, while in the left eye shows loss of ellipsoid zone, macular thinning with reverse shadowing. (**G**,**H**) are Goldman visual fields of both eyes showing normal visual field in the right eye and a large central scotoma in the left eye.

Best-corrected visual acuity (BCVA) was 20/20 and hand movements (HM) were positive in the right and left eye, respectively. Anterior segment examination of both eyes, and fundus examination of the right eye were unremarkable apart from early cataractous changes in both eyes. Fundus examination of the left eye revealed midperipheral bone spicules, localized retinal thinning nasal to the optic disc, attenuated blood vessels, a waxy pallor of the optic disc and atrophic macula with pigment clumping (Figure 3).

Fundus autofluorescence revealed a large hypoautofluorescent ring in the posterior pole surrounding the disc and macula, and a hyperautofluorescent ring at the macular area with patches of well demarcated hypoautofluorescence. The SD-OCT of the right eye was unremarkable, while there was significant loss of the ellipsoid zone and macular thinning with reverse shadowing in the left eye. ffERG was non-recordable in the right eye with profoundly reduced photopic and scotopic responses in the left eye including a delayed 30 Hz flicker (Figure 3).

The Goldmann visual field showed a full field with an enlarged blind spot with object I3e and an inferotemporal wedge defect in the right eye, while the left had a large central scotoma. Next-generation sequencing for cone–rod dystrophy genes revealed a hemizygous missense c.1286 C>T (p.Pro429Leu) variant in the *RPGR* gene (NM_000328.2). To the best of our knowledge, this mutation has not been described in the literature. The allele frequency of this variant has not been documented in the normal population (GnomAD v2.1.1 controls; accessed on August 15, 2020). Seven out of eleven bioinformatic in silico programs predicted pathogenicity of the hemizygous c.1286 C>T (p.Pro429Leu) variant. Segregation analysis of other family members was not possible.

The patient had stable vision and examination in both eyes during six years of follow-up visits with stable average central macular thickness on SD-OCT (Table 1).

## 4. Discussion

We herein describe two patients with URP and possible ciliopathy, associated with two novel variants in *AGBL5* and one novel variant in *RPGR*, respectively. These genes have not been previously associated with URP.

Francois and Verriest set up a specific set of criteria to diagnose URP, which includes (1) the exclusion of all other etiologies that might cause pseudo retinitis pigmentosa, (2) the presence of typical clinical signs of retinitis pigmentosa in one eye only, (3) no symptoms or signs and a normal electroretinographic finding in the fellow eye and (4) a long follow-up duration for at least 5 years to rule out the possibility of asymmetric inherited RP [14].

While it has been suggested that all of these criteria are necessary to diagnose URP [15], the 5-year follow-up duration to diagnose URP today seems to be an unnecessarily long follow-up period in the era of advanced molecular genetic testing. In patient 2 the ffERG was affected in both eyes, demonstrating a bilateral retinal dystrophy. Similarly, Errera et al. reported 42 patients with URP, where eight patients had subnormal ffERG findings in the fellow eye [4]. The chorioretinal atrophic changes in the peripapillary area along with increased autofluorescence in the right eye of patient 2 might indicate an asymmetric presentation of a bilateral RP.

The *AGBL5* (ATP/GTP-binding like protein) gene has recently been found to cause autosomal recessive non-syndromic retinitis pigmentosa (arRP) [15,16,17]. *AGBL5* is a member of the cytosolic carboxypeptidase (CCP) protein family; members of this family are involved in post-translational modification (PTMs) of a- and b-tubulin, which are the main constituents of microtubules. The human retina shows *AGBL5* immunoreactivity in all layers and most prominently in the cone’s inner segment, ganglion cells and nerve fiber layer. *AGBL5* is known to function in the polyglutamylation and deglutamylation of tubulin, which can affect tubulin function or the binding pattern. The connecting cilium of the photoreceptor is crucial for outer segment renewal and for the transport of metabolites between the outer and inner segment, and it contains tubulin. Therefore, perturbations in this pathway may lead to ciliopathy and photoreceptor cell degeneration [18,19]. One study by Pathak et al. reported that cytosolic carboxypeptidases were found to increase cilia tubulin glutamylation in zebrafish, which may lead to ciliogenesis; therefore, we speculate that *AGBL5* mutation might cause ciliopathy in humans through affecting the tubulin component of human cilia [20].

*RPGR* gene (retinitis pigmentosa GTPase regulator) has been found to cause X-linked retinitis pigmentosa (XLRP) [21]. Moreover, *RPGR* mutations have been associated with X-linked cone–rod dystrophy and X-linked macular degeneration with normal electroretinographic findings [22]. The gene is located on the short arm of the X chromosome; it has been found to be expressed in vertebrate tissues such as in the eye, brain and kidneys. The two major isoforms in the eye are RPGR EX1-19 and RPGR ORF16, and they are mainly confined to the connecting cilia and photoreceptor outer segment [21].

The exact function of *RPGR* is still unclear, but it is thought to be a putative guanine nucleotide exchange factor for an unknown G protein [23]. The *RPGR* gene products are associated with the centrosomes and colocalize with microtubules of the ciliary axoneme, which are related to the connecting cilium of photoreceptors [24]. There are four ciliary compartments in the photoreceptor: the distal cilium, connecting cilium or proximal cilium, the basal body and periciliary complex [8]. The connecting cilium acts as a diffusion barrier between the inner segment and outer segment for the bidirectional transport of a large amount of proteins including opsin, rhodopsin, transducin and other essential proteins for phototransduction. The inner segment and outer segment have different protein compositions and functions. The inner segment carries all organelles for metabolic function, while the outer segment is a specialized organelle where the phototransduction cascade takes place. The outer segment is continually regenerated throughout the life and maintenance of the outer segment, crucial for the efficient phototransduction and long-term survival of the photoreceptors. Therefore, disruption in the connecting cilium function or structure may result in significant photoreceptor dysfunction [20,21,22,23].

To the best of our knowledge, neither *RPGR* or *AGBL5* mutations have been implicated previously in URP (Table 3) [25].

In this report, one of our patients (patient 2) showed an abnormal hyperautofluorescent ring, which is one of the structural changes often seen in RP associated with ciliopathy. On the other hand, both patients showed some changes on the EZ line on SD-OCT. In patient 1 with *AGBL5* variants, the parafoveal EZ was barely visible and the subfoveal EZ was minimally affected. On the other hand, in patient 2 with the *RPGR* variant, the EZ was severely affected and barely visible. Interestingly, both cases have mutations that may lead to defective intracellular transportation between the photoreceptor’s inner and outer segments and dysfunction of the photoreceptor-connecting cilium, and may eventually cause RP associated with ciliopathy.

As a limitation of this study, segregation analysis of these variants was not possible. Further work in the future is needed to strengthen the observation that these variants might be disease-causing and associated with URP with possible ciliopathy.

## Figures and Tables

**Table 1:** Characteristic findings that differentiate URP from other differential diagnoses.

Autoimmune Retinopathy/Cancer-Associated Retinopathy	Inflammatory Disease	Congenital Infection	Unilateral Retinitis Pigmentosa	Diagnosis
Positive history of systemic autoimmune diseases or neoplasms	History of eye redness or floaters or reduced vision	Maternal infection during pregnancy. Systemic manifestations of the disease	Positive family history can be found	History
Pigmentary retinopathy, can be normal examination	Anterior chamber or vitreous cells, anterior or posterior synechiae. Retinitis, vasculitis	Cataract, posterior synechiae, strabismus, retinal pigments	Unilateral bone spicules, attenuated vessels and pale optic disc	Clinical findings
Reduced photopic ± scotopic responses	Usually normal	Reduced photopic ± scotopic responses	Reduced scotopic ± photopic responses	Electroretinography
Positive antiretinal antibodies	Positive uveitis workup	Positive serologic testing for TORCH (toxoplasmosis, others (syphilis), rubella, cytomegalovirus, herpes)	Positive genetic testing	Lab/additional investigations

**Table 2:** Serial visual acuity and serial central macular thickness measurements by spectral domain optical coherence tomography (SD-OCT) in 2 patients with unilateral retinitis pigmentosa. Both patients had stable vision and central macular thickness throughout the follow-up.

	Patient 1 (Two Heterozygous Variants c.373 C>T (p.Arg125Trp) and c.730-22_730-19 dup in *AGBL5*				Patient 2 (*RPGR* Missense Variant c.1286 C>T (p.Pro429Leu)			
Year	VA OD	VA OS	CMT OD	CMT OS	VA OD	VA OS	CMT OD	CMT OS
**2015**	NA	NA	NA	NA	20/20	HM	285 μm	263 μm
**2016**	20/20	20/40	287 μm	285 μm	NA	NA	NA	NA
**2017**	20/20	20/40	278 μm	285 μm	NA	NA	NA	NA
**2018**	NA	NA	NA	NA	NA	NA	NA	NA
**2019**	20/20	20/40	271 μm	275 μm	20/20	HM	274 μm	255 μm
**2020**	NA	NA	NA	NA	20/20	HM	262 μm	245 μm

VA = visual acuity, CMT = central macular thickness, NA = not analyzed, HM = hand motions, OD = right eye, OS = left eye, um = micrometers.

**Table 3:** Unilateral RP cases in the literature confirmed by genetic testing.

Authors	Age, Gender	Eye, Symptoms	Initial VA	Retinal Examination	ERG	VF	VA	Genetics
Makhupadhyay et al. 2011 [6]	63, F	Right, asymptomatic	20/30	Bone spicules, attenuated vessels, pale disc	Flat scotopic and markedly reduced photopic	Mildly constricted	N/A	*RP1* p.R677X heterozygous nonsense mutation
Marsiglia et al. 2013 [5]	8, F	Right, nyctalopia	20/20	Bone spicules, attenuated vessels, pale disc	Markedly reduced scotopic and photopic	N/A	N/A	*USH2A* Trp4149Arg
Mercado et al. 2018 [25]	15, F	Left, nyctalopia	20/25	Bone spicules, attenuated vessels, pale disc	Flat scotopic and photopic	N/A	20/25	*USH2A* gene c.6958-5 C>T (intronic splice variant) and c.6638T.A (Val2228Glu) (missense variant))
Sim et al. 2018 [7]	12, F	Right, asymptomatic	6/5	Bone spicules, attenuated vessels, pale disc	Flat scotopic and photopic	Severely constricted	6/9	*CLRN1* heterozygous mutation c.118T>G (p.Cys40Gly)

VA = visual acuity VF = visual field.

## Data Availability

The work was conducted at King Khaled Eye Specialist Hospital (KKESH) and all related data are available at KKESH.
